# Reflections on 10 years of research capacity building

**DOI:** 10.1038/s41533-026-00484-8

**Published:** 2026-05-21

**Authors:** Gwyneth A. Davies, Hilary Pinnock, Kathryn Ferris, Aziz Sheikh

**Affiliations:** 1 Professor of Respiratory Medicine, Population Data Science, Swansea University Medical School, Swansea, UK; 2 Professor of Primary Care Respiratory Medicine, Usher Institute, The University of Edinburgh, Edinburgh, UK; 3 ST8 Paediatric Doctor, Royal Belfast Hospital for Sick Children, Belfast, UK; 4 Head of Department, Nuffield Department of Primary Care Health Sciences, Oxford, UK

**Keywords:** Education, Environmental social sciences

## Abstract

There are major concerns about the decline in translational research capacity in the UK with potential knock-on implications for advances in clinical care and the growth of the life sciences sector. As co-leads and student representative for a UK-wide Postgraduate Training and Capacity building programme for the Asthma UK Centre for Applied Research (AUKCAR), we have learnt much from our 10 years of developing and delivering a UK-wide academic training initiative involving 17 partner universities. We discuss learnings from a broader research capacity building perspective, with a focus on maintaining a pipeline of respiratory-interested researchers, including clinical academics. In this article, we reflect on what we hoped to achieve, the lessons learnt along the way, and implications for others seeking to improve the research pipeline.

Historically, the UK has been at the forefront of biomedical science globally, with world-renowned academic research institutions, the unmatched research potential of the NHS and a vibrant life sciences industry. A mounting financial crisis affecting UK universities threatens UK research capacity. Reports by the Medical Research Council (MRC)^1^ and the Academy of Medical Sciences^2^ have sounded the alarm for the future of health research in the UK; these highlight recent issues challenging the sector which include increased international visa fees and Brexit, alongside a cost-of-living crisis and relatively uncompetitive salaries^1–4^. Moreover, on average, the UK spends significantly less on research and development (R&D) than many other countries. Office for National Statistics (ONS) data, for example, reveal a drop in the level of investment in R&D by £80 million in real terms between 2021 and 2022^5^.

A survey by Universities UK estimates the net impact of government policy decisions will be a £1.4 billion reduction in funding to higher education providers in England in 2025–26^6^. This means that universities may struggle to afford to train the next generation of researchers. In fact, the PhD is under threat globally – with countries including France, Germany, Italy, the Netherlands, Australia, South Africa, Japan, Brazil, Canada, and the UK all reporting a fall in PhD researchers in recent years^7^. In the UK, the academic job market for PhD graduates is also shrinking.

Focusing specifically on clinical research, a recent MRC report^1^ evaluated the decline in UK clinical researchers and identified necessary actions at a national level. A similar report by the Office for Strategic Coordination of Health Research (OSCHR) found this to be a multi-disciplinary issue^8^. Research active hospitals have better patient outcomes, and medical research is crucial to growing the economy; every £1 invested delivers a further ~25p return for every year thereafter^9^. The COVID-19 pandemic is a compelling example of the UK’s illustrious history of research driving innovation, from understanding the virus, to the RECOVERY trial^10^ identifying the first effective treatment, and development of novel vaccines. Key to this world-leading endeavour was the balance of discovery, clinical and public health research across academic, healthcare and industry settings underpinned by cross-sectoral collaborative partnerships and patient and public involvement and engagement (PPIE).

Future applied research is threatened by a decline in clinical research staff over the last decade, both in absolute numbers and, more importantly, in proportion to the expanding NHS workforce. Lord Darzi previously highlighted that clinical research drives innovation, which improves NHS sustainability and leads to better patient outcomes^11^. Although the recent NHS 10-Year Plan^12^ and the Life Sciences Sector Plan^13^ shows commitment to strengthening the economic and healthcare value of research, the UK is still not adequately growing its clinical research capability in parallel with NHS expansion and the increasing clinical need.

## Addressing the decline in research capacity building

Tackling this decline is essential to the long-term sustainability of the NHS and to broader economic growth, enabling patients to benefit from medical innovation and securing the UK’s position as a leader in biomedical science. Recommendations included in the recent MRC report^1^ are to establish a common national clinical research career framework and to provide visible leadership and mentorship delivered by established researchers.

Echoing these goals, the Asthma UK Centre for Applied Research (AUKCAR) was established to develop much-needed capacity in applied asthma research^14^. As co-leads and student representative for the AUKCAR Training and Capacity building programme, we have learnt much from our 10 years (2015–24) of developing and delivering a capacity building initiative involving 17 partner universities working with NHS providers. Specifically, our programme supported 20 clinical respiratory PhDs and early career researchers (ECRs) and 39 applied non-clinical PhDs and ECRs, contributing to reversing the decline in clinical research staff, and the threat to the growth of the life sciences sector^1^. We have a number of key lessons to share which may be valuable to others supporting national – or potentially global – research capacity building initiatives.

Creating a pipeline of respiratory-interested researchers was a joint goal of AUKCAR. We grew capacity from core funding for 21 studentships and ECRs to having supported and trained over 50 doctoral students and ECRs over 10 years, through a highly interdisciplinary cross-institutional approach, which was appreciated by students and affirmed the UK-wide collaboration. Our experience of nurturing a tranche of PhD students was generally positive with critical elements of the successful programme being transferrable to other specialties.

## What have we learned in 10 years

Our blended doctoral training programme included mandatory cross-institutional supervision to underscore the importance of team science (Table 1). Formal evaluation throughout the programme showed that this model worked well in fostering new collaborations and facilitating delivery of PhD projects (such as ‘second coders’ or ‘duplicate reviewers’). Feedback shaped the training, such as funding a virtual writing retreat after this was highlighted by students. The common purpose proved important in developing collaboration across our UK-wide Centre.

**Table 1:** Lessons learnt for developing a UK-wide doctoral^a^ and post-doctoral^b^ training programme to support research capacity building.

Innovation	Reflections on implementing innovation
Cross-institutional supervision^a^	Team science approach, external supervisor identified by main supervisor: providing additional training resources, fostering new interdisciplinary collaborations, facilitating projects like systematic reviews.
Embedding PPIE^15^ from outset^a,b^	Students appreciated learning from patients; conversely people living with asthma enjoyed supporting ‘their’ student.
Peer mentorship and peer-led^a^ learning^18,19^	Diverse range of shared experiences and perspectives. Fostering resilience. Personal development and developing a ‘researcher identity’.
Creating a whole research community^a,b^	Being part of and being valued by something bigger: providing a sense of community and belonging. Affiliation of students and ECRs, achieving a critical mass^16^. Cohesive and supportive student network: social opportunities to foster long term relationships.
Leadership opportunities^b^	Supporting fellowship applications, small grant proposals, co-applicants on grants, undergraduate and masters-level supervision. Nurturing future academic and clinical leaders.

Patient and public involvement and engagement (PPIE) was embedded in doctoral projects from the outset. PPIE is a critical component of high quality research and integrating the lived experience was a requirement of all the AUKCAR doctoral projects^15^. PPIE colleagues reviewed PhD proposals and sat on interview panels, contributing to student selection. PPIE was new to many students and PPIE-led training sessions enabled greater understanding of its importance. Formal feedback showed that this PPIE contribution was highly valued by students, but anecdotally was also appreciated by PPIE colleagues who took an on-going interest in the progress of ‘their student’, a relationship that we would plan to capture more formally in future programmes. PPIE colleagues have reflected on their role as educators on our programme^15^.

We encouraged affiliation of students and ECRs beyond those funded by AUKCAR to include those working in aligned research, therefore growing the training opportunities and enabling an inclusive culture within the Centre. A critical mass was thus achieved, aligning with the Salzburg principles which underpin doctoral education in Europe^16^.

Our students valued being part of a UK-wide Centre which offered the combination of personalised student support and wider opportunities that included contributing to multidisciplinary research, access to professional development and widening career prospects with expert trainers identified across partner universities and externally e.g. presentation skills. The virtual aspect of our doctoral programme helped with resilience during the pandemic, providing a safe and inclusive network for students during a particularly turbulent time.

As our first students graduated in 2019, we focused on establishing a programme for our own and other ECRs. It soon became clear that this needed a different model to our doctoral programme due to the nebulous nature of ECRs at different career stages. Formal training was less valued, and for future programmes, we would concentrate on supporting fellowship applications, creating opportunities to develop small grant proposals, and serve as co-applicants on large grants, and developing undergraduate and masters-level supervision and teaching opportunities. We formed an alumni network and found that over 80% of researchers were retained in research with three securing posts in industry. Crucially, many of the clinical researchers went on to secure clinical academic posts. For future programmes, we would also aim to support alternative routes within the wider research infrastructure – e.g. Clinical Trials Units – and to capture further employment metrics.

The launch of the new Centre for Applied Respiratory Research, Innovation, and Impact (CARRii) will build on our learning. Recent changes to capacity building within National Institute for Health and Care Research (NIHR) programmes could be harnessed to support our approach^17^. Adopting the established focus on research capacity building in global health grants, NIHR Programme Grants for Applied Research now offer a formal remit to build and support research careers. Our experience suggests that this will be a valuable approach as, for example, the AUKCAR funded two of three PhD studentships aligned with the IMPlementing IMProved Asthma self-management as RouTine (IMP^2^ART) programme (https://usher.ed.ac.uk/imp2art/student-involvement) – an arrangement that offered the students the experience of being part of a large research team as well as enhancing the programme of work.

Doctoral training programmes and nurturing ECR career development, especially supporting clinical academics, are crucial to the future of medical science globally. Our experience suggests that a UK-wide capacity building programme, underpinned by a highly collaborative network and united by common goals, could contribute an innovative solution to the current challenges.

## Data Availability

No datasets were generated or analysed during the current study.
